# Exploration of the Mechanism of Tongmai Xianxiang in Treating Postherpetic Neuralgia Based on Gas Chromatography–Mass Spectrometry Analysis and Network Pharmacology

**DOI:** 10.1155/ijog/7415848

**Published:** 2025-10-21

**Authors:** Linglong Qu, Hui Wang, Yufeng Huang, Zhen Gao, Ruichang Song, Shiyu Sun, Pingping Shao, Rukai Wang, Chunlin Li

**Affiliations:** ^1^ Department of Internal Medicine of Traditional Chinese Medicine, First School of Clinical Medicine, Shandong University of Traditional Chinese Medicine, Jinan, Shandong, China, sdutcm.edu.cn; ^2^ Department of Acupuncture-Moxibustion, Zaozhuang Hospital, Dongfang Hospital of Beijing University of Chinese Medicine, Zaozhuang, Shandong, China, bucm.edu.cn; ^3^ Department of Pharmacology, School of Pharmaceutical Sciences, Cheeloo College of Medicine, Shandong University, Jinan, Shandong, China, sdu.edu.cn; ^4^ Department of Traditional Chinese Medicine, College of Traditional Chinese Medicine, Shandong University of Traditional Chinese Medicine, Jinan, Shandong, China, sdutcm.edu.cn; ^5^ Department of Neurology, Affiliated Hospital of Shandong University of Traditional Chinese Medicine, Jinan, Shandong, China, sdutcm.edu.cn

**Keywords:** Chinese medicine Tongmai Xianxiang, gas chromatography–mass spectrometry, molecular docking, network pharmacology, postherpetic neuralgia

## Abstract

**Objective:**

The aim of this study is to explore the potential target of Tongmai Xianxiang in the prevention and treatment of postherpetic neuralgia (PHN) by gas chromatography–mass spectrometry (GC‐MS) and network pharmacology.

**Methods:**

Quantitative analysis using GC‐MS was employed to obtain the chemical components of Tongmai Xianxiang, and target site prediction was conducted; PHN‐related targets were collected from relevant disease databases; the anti‐PHN targets of Tongmai Xianxiang were obtained through network pharmacology methods, and molecular docking was performed on the main targets.

**Results:**

A total of 633 active components were screened by GC‐MS, corresponding to 772 targets. By searching the disease database, a total of 169 PHN‐related disease targets were obtained after removing duplicates. A total of 53 core genes, including SLC6A4, MAOA, DRD2, and ALB, were obtained by intersecting the corresponding target and PHN target. Through molecular docking, the results indicate that ALB has the lowest free binding energy with Z‐butylidenephthalide, 2‐undecanol, and 1,6,10‐dodecatrien‐3‐OL, 3,7,11‐trimethyl‐, (E)‐.

**Conclusion:**

Tongmai Xianxiang can prevent PHN by multitarget, multiprocess, and multipathway. This study provides new ideas and new targets for the intervention of Tongmai Xianxiang in PHN.

## 1. Introduction

Herpes zoster is a blistering skin disease caused by varicella–zoster virus infection. After infection, the virus invades and lies dormant in the body′s ganglia. When the immune function of the human body decreases, the virus can be reactivated, leading to a secondary recurrence of herpes zoster after treatment [1–3]. Postherpetic neuralgia (PHN) is caused by the herpes zoster virus invading nerve cells, triggering inflammatory reactions or neuronal necrosis, which leads to persistent pain. The most common complication of herpes zoster is PHN, characterized by persistent, spontaneous, and locally intense pain. Most patients experience hyperalgesia or even allodynia [4, 5]. The incidence of PHN in the population is approximately 3.9–42.0 per 100,000 individuals [3, 6, 7]. Studies [7] indicate that about 29.8% of patients with herpes zoster develop PHN, with the incidence increasing with age. PHN significantly worsens patients′ pain and impacts their physical and mental health. Therefore, clinical treatment focuses on promptly managing pain; reducing the recurrence rate of PHN; alleviating sleep disturbances, anxiety, and depression caused by pain; and enhancing patients′ quality of life [6, 8].

Currently, promoting nerve repair and regulating nerve function are primary focuses in the treatment of PHN. Treatment methods include drug therapy and minimally invasive interventional therapy, with drug therapy being the most basic and commonly used approach [2, 9]. The recommended first‐line drugs for PHN are calcium channel modulators such as pregabalin and gabapentin, as well as tricyclic antidepressants like imipramine and clomipramine [10–15]. Additionally, lidocaine patches can be applied for local anesthesia and analgesia [16, 17]. However, drug treatment often leads to various adverse reactions. Prolonged use of these drugs can cause gastrointestinal issues, immune dysfunction, and side effects such as drowsiness, fatigue, and constipation. Drug treatment also shows inconsistent efficacy and may result in disease recurrence. Nondrug treatments mainly include nerve block anesthesia and nerve damage treatments. But the nerve damage treatment is irreversible and may increase the incidence of abnormal pain while potentially causing complications in the affected nerve area. In contrast, traditional Chinese medicine offers a variety of external treatments for pain, effectively reducing patient reliance on oral medications. This approach has gained widespread use in clinical treatment and is attracting increasing attention [18, 19].

Aromatherapy refers to the medical and healthcare method of converting aromatic substances into a linear aroma and inhaling this aroma after ignition [20]. This practice aims to prevent and treat diseases and promote health. Aromatic substances act on the human skin and mucous membranes through inhalation and direct contact [21–25]. They disperse throughout the body and penetrate the viscera, thereby balancing yin and yang, enhancing healthy qi, and resisting pathogenic factors. Aromatherapy is noted for its minimal side effects, painless comfort, simple operation, and affordability, making it a promising treatment option for PHN in clinical settings.

Clinical aromatherapy has been found beneficial in the inpatient and outpatient settings [26, 27]. Two meta‐analyses have shown that aromatherapy was performed as a supportive analgesic method during labor [28, 29]. Studies have shown that aromatherapy effectively reduces pain, anxiety, and stress levels in palliative care patients and improves their quality of life [30]. Studies have confirmed that aromatherapy plays a role in migraine treatment by effectively inhibiting neurogenic inflammation, hyperalgesia, and balancing vasorelaxation [31]. From this, it can be seen that aromatherapy, as an alternative treatment method, has proven effective in various clinical scenarios such as pain, anxiety, and insomnia. This provides strong evidence for the use of aromatherapy in treating PHN.

In traditional Chinese medicine, the main pathogenesis of PHN is summarized into the following two aspects: First, the circulation of qi and blood is blocked, and when circulation is disrupted, pain occurs. Second, the disease persists for a long time, resulting in blood deficiency and an inability to nourish the meridians, and the deficiency of the meridians leads to pain.

In this study, a self‐made traditional Chinese medicine, Tongmai Xianxiang, which was tested by NTEK (Report Number: NTEK‐SC09211708170305C), indicates that the elements that can migrate and the harmful substances after combustion of this incense are all qualified, and a Chinese national invention patent has been applied for (Patent Number: ZL202410730937.8). It was used to finely crush components such as Gangbangui, Dahuang, Ruxiang, Moyao, Sanleng, and Tanxiang. Approximately 1 g of musk was added per kilogram of traditional Chinese medicine powder. After thorough mixing, combustion improvers and adhesives were added to facilitate shaping, and the mixture was sealed and preserved after molding. In Tongmai Xianxiang, Gangbangui clears heat and detoxifies. Ancient prescriptions mention using fresh Gangbangui and realgar powder to treat what is now called herpes zoster. Dahuang, bitter and cold, cools blood, purges fire, and detoxifies. Ruxiang, Moyao, and Sanleng promote blood circulation and remove stasis, while Tanxiang promotes qi and relieves pain. According to traditional Chinese medicine theory, Tongmai Xianxiang utilizes musk′s heat to enhance the efficacy of these herbs against disease. The Tongmai Xianxiang is used to treat the pain symptoms caused by PHN. It lays the foundation for the treatment of the underlying causes of the patient′s pain relief. This is also in line with the traditional Chinese medicine principle of “treating the symptoms urgently when they are severe and addressing the root cause when they are mild.” The selected herbs in Tongmai Xianxiang synergize to promote blood circulation, detoxify, remove stasis, open meridians, promote qi, and alleviate pain.

The internationally recognized and most direct analytical target in the field of aromatherapy research is the volatile organic compounds released by it. The gas chromatography–mass spectrometry (GC‐MS) analysis selected in this study accurately identified the active molecules that can be absorbed, which is a standard and mature research method in aromatherapy. Many high‐quality studies have adopted this approach [32–35]. Network pharmacology is an interdisciplinary field combining pharmacology and biology theories. This paper employs GC‐MS analysis and molecular docking technology to uncover the network interactions between Tongmai Xianxiang and PHN. The volatile components of Tongmai Xianxiang were analyzed to investigate their targets in treating PHN and predict the potential pharmacological mechanisms. These findings aim to offer new insights for the research and development of novel drugs and the external treatment of traditional Chinese medicine.

## 2. Materials and Methods

### 2.1. Meteorological Chromatography–Mass Spectrometry Analysis

#### 2.1.1. Materials and Instruments

Tongmai Xianxiang is a self‐made prescription, and the raw material is sourced from Hebei Hongmeng Xiangye Co. Ltd., with products that have successfully undergone CMA safety testing and certification. The solid phase microextraction fiber (SPME) used, coated with divinylbenzene/carboxen/polydimethylsiloxane (DVB/CAR/PDMS, 50/30 *μ*m × 1 cm), was acquired from Supelco (Bellefonte, United States). Ethanol was procured from Aladdin (Shanghai, China), while N‐hexyl‐d13 alcohol came from C/D/N Isotopes Inc. (Quebec, Canada). Saturated alkanes was purchased from Sigma (United States), and N‐hexane was purchased from Yonghua (Shanghai, China). H_2_O was sourced from Watsons (Guangzhou, China).

#### 2.1.2. Experimental Steps and Chromatographic Mass Spectrometry Conditions

To closely simulate the effects of Tongmai Xianxiang on the human body, GC‐MS was chosen to capture the volatile active substances that influence human physiology postadministration of Tongmai Xianxiang. GC‐MS is preferred over liquid chromatography–mass spectrometry (LC‐MS) due to its superior accuracy in identifying and quantifying metabolites. The specific experimental procedures are outlined as follows:

Solution preparation: A suitable amount of deuterated n‐hexanol‐d13 was dissolved in a 50% ethanol aqueous solution and diluted to 1 mg/L to create a standard solution, which was stored in a refrigerator at 4°C. Then, 1000 mg/L n‐alkanes were taken and stepwise diluted with n‐hexane to prepare a 1 mg/L solution. The reserve solution was also stored in a refrigerator at 4°C.

Extraction of active volatile substances: 0.5 g of the sample was accurately transferred into a 20 mL headspace sampling bottle. Subsequently, 10 *μ*L of internal standard solution was added, and the mixture was incubated at 60°C for 30 min. Following this, the sample was aged at 270°C for 10 min. Next, the SPME fiber was moved to the incubation chamber, where it adsorbed the volatile substances from the sample at 60°C for 30 min. After adsorption, the SPME extraction head was transferred to the GC (gas chromatography) inlet and desorbed at 250°C for 5 min. The temperature was then adjusted to 270°C and maintained for 10 min to ensure complete desorption and aging. Subsequently, 10 *μ*L of n‐alkanes was transferred to a 20 mL headspace injection bottle for incubation, extraction, and injection. Once all samples were processed in this manner, GC‐MS analysis was conducted to obtain the final results.

Mass spectrometry conditions: The LECO Pegasus BT 4D mass spectrometry detector was used with a transmission line temperature of 250°C and an ion source temperature of 250°C. The acquisition rate was set to 200 spectra per second. Electron bombardment at 70 eV was used as the ionization source, with a detector voltage of 2016 V. The mass spectrometry scanning range was configured from *m*/*z* 35 to 550.

#### 2.1.3. Identify the Relative Content of Each Component of the Volatile Components

The total ion current chromatogram of Tongmai Xianxiang was obtained through GC‐MS analysis. The ChromaTOF software processed the comprehensive two‐dimensional GC data using the NIST2020 database.

### 2.2. Target Prediction of Active Components of Tongmai Xianxiang

The active ingredients and their relative contents were determined through GC‐MS analysis. The 2D structure of each active ingredient was retrieved from PubChem (https://pubchem.ncbi.nlm.nih.gov/), and the Swiss Target Prediction website (http://www.swisstargetprediction.ch/) was used to predict their target proteins. Any ingredients that did not match the molecular formula or CAS number were excluded to identify the effective compounds in Tongmai Xianxiang.

### 2.3. Acquisition of Targets Related to PHN

The databases DisGeNET, GeneCards, TTD, DrugBank, and OMIM were searched using “postherpetic neuralgia” as the keyword to gather targets associated with PHN. The targets obtained were standardized and named using the UniProt website. Targets related to PHN were identified after eliminating duplicates.

### 2.4. Acquisition of Common Targets

Using the Venn diagram method, the common targets of the active components between the Tongmai Xianxiang and PHN were obtained. The subsequent series of bioinformatics analyses are all based on the common targets of Tongmai Xianxiang and PHN.

### 2.5. Component–Disease–Target Network Construction

Based on the Cytoscape 3.9.1 software, nodes representing Tongmai Xianxiang, active ingredients, diseases, and intersection targets were selected. Topological analysis was conducted using Network Analyzer, calculating the degree, betweenness centrality (BC), and closeness centrality (CC) for each node. The median values of BC and CC were taken as thresholds. Key active ingredients were ranked based on their degree values and screened accordingly.

### 2.6. Construction of Protein–Protein Interaction (PPI) Network

The potential targets of Tongmai Xianxiang for preventing and treating PHN were uploaded to the STRING database (https://cn.string-db.org/). Screening criteria were set with a score threshold of > 0.4. The retrieved data were then imported into Cytoscape 3.9.1 software to construct a PPI network diagram. The core target for treating PHN was identified using Network Analyzer.

### 2.7. Gene Ontology (GO) Functional Enrichment and Kyoto Encyclopedia of Genes and Genomes (KEGG) Pathway Analysis

The intersection targets were input into the DAVID database (https://david.ncifcrf.gov/) for GO function enrichment analysis and KEGG pathway enrichment analysis. Set FDR < 0.05 as the screening threshold. FDR is the value obtained after multiple tests on *p* values, which can reduce the false positive rate. After excluding nonspecific GO functions and KEGG pathways, select the items with larger FDR values to identify those that have a higher correlation with PHN. Results closely associated with PHN were selected, and a bubble map was generated using the Weishengxin platform (https://www.bioinformatics.com.cn). Using Cytoscape 3.9.1 software, a Tongmai Xianxiang–target–pathway–disease network was constructed to predict the potential biological targets and pathways relevant to PHN.

### 2.8. Molecular Docking Verification

The components ranked highest by degree value in the network diagram were identified as the primary active components of Tongmai Xianxiang. Their small‐molecule ligands were then subjected to molecular docking with the core proteins in the PPI network. The 2D structure of these small‐molecule ligands was retrieved from the PubChem database (https://pubchem.ncbi.nlm.nih.gov/) and converted into PDF format files using OpenBabel software. The best 3D crystal structure files of proteins were obtained by integrating data from the STRING and PDB databases, saved in PDF format. AutoDock software was employed for molecular docking to calculate the minimum binding energy between each target and its corresponding active ingredient. Finally, the results of molecular docking were visualized and verified using PyMOL software.

## 3. Results

### 3.1. Qualitative and Quantitative Results of GC‐MS

Figure 1 depicts the total ion current diagram obtained from the detection of volatile components in the traditional Chinese medicine Tongmai Xianxiang.

Figure 1Total ion current chromatograms: (a) one‐dimensional, (b) two‐dimensional, and (c) three‐dimensional. The abscissa is the retention time (seconds), and the ordinate is the ionic strength. The color represents the ion peak of the detected substance. The redder the color, the higher the response intensity.

**Figure:**
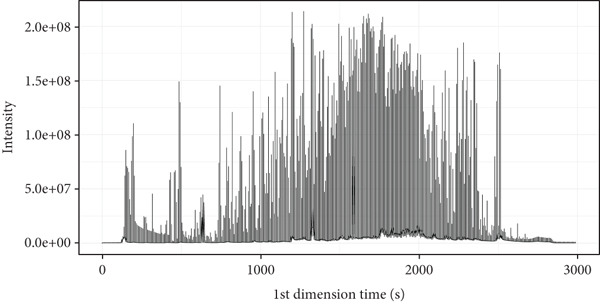
(a)

**Figure:**
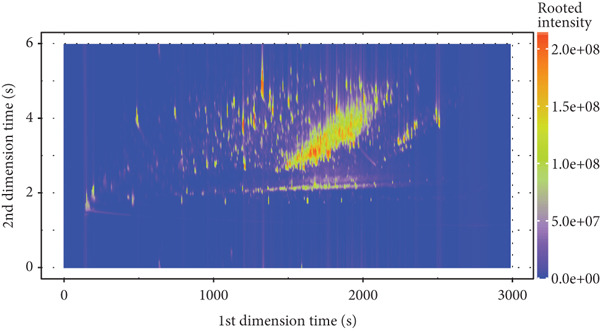
(b)

**Figure:**
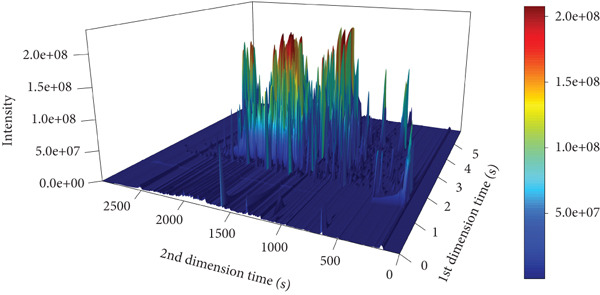
(c)

The volatile components and their respective percentage content were identified through analysis of the total ion chromatogram. To facilitate comparison of data across different magnitudes, normalization techniques such as total peak area normalization or internal standard normalization were applied to the raw data.

**Figure 2:**
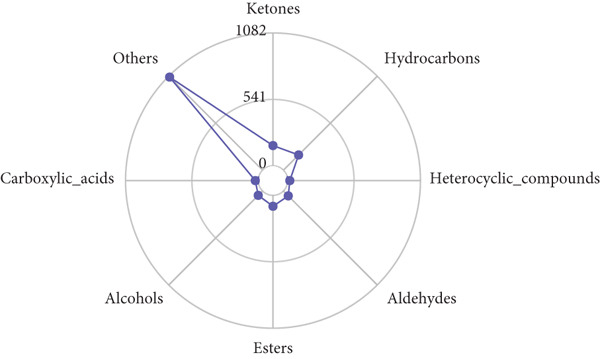
Statistical radar chart of the number of material species. The outermost ring name represents the type of substance, and the broken line represents the number of flavor substances corresponding to the type.

A total of 946 compounds were isolated from the volatile components of Tongmai Xianxiang. After excluding substances with unclear composition or structure, or those with a difference between the actual retention index and the reference retention index ≥ 20, 567 compounds were retained. These include 197 types of esters, 102 types of benzene derivatives, 77 types of organic heterocyclic compounds, 60 types of hydrocarbons, 57 types of ketones, 38 types of aldehydes, 19 types of alcohols, 11 types of other organic oxygen compounds, five types of organic acids and their derivatives, and one type of organic sulfur compound. Establish a statistical radar map of the number of material types, as shown in Figure 2.

### 3.2. Effective Components and Targets of Tongmai Xianxiang

The qualitative and quantitative screening results from GC‐MS were imported into the PubChem database to obtain SMILES numbers and 2D molecular structures of active substances. Through SMILES number matching in the Swiss Target Prediction database, 7297 efficacy targets were identified. After deweighting, 772 drug targets were retained.

### 3.3. Screening of Targets for PHN

**Figure 3:**
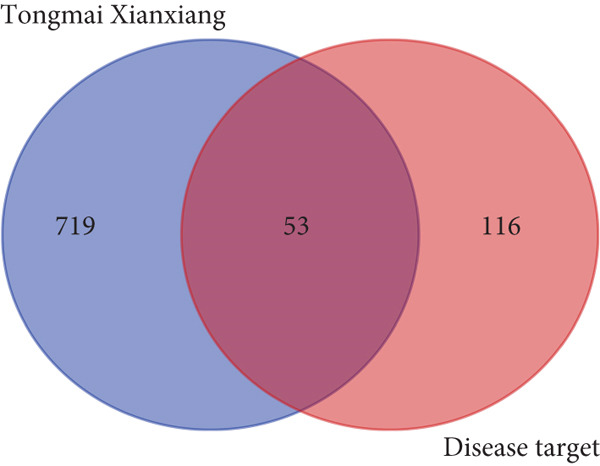
Venn plot of Tongmai Xianxiang–disease.

PHN‐related disease targets were searched across DisGeNET, GeneCards, TTD, DrugBank, and OMIM databases, resulting in the identification of 169 PHN‐related targets after filtering. By comparing these targets with those of Tongmai Xianxiang, 53 intersection core targets were identified (Figure 3).

### 3.4. Network Construction

**Figure 4:**
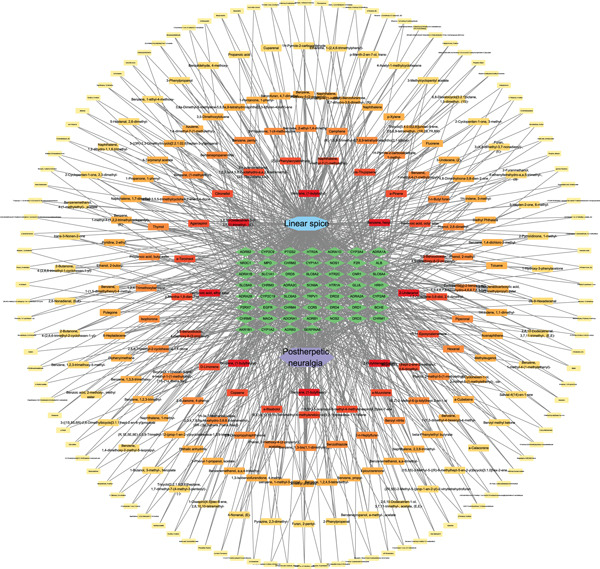
Tongmai Xianxiang–active ingredient–target–disease network diagram.

Cytoscape 3.9.1 was utilized to construct two networks: the Tongmai Xianxiang–active ingredient–target–disease network (Figure 4).

Considering that Tongmai Xianxiang′s composition is derived from the burning of natural Chinese medicine substrates, which closely mimics its action in the human body, this article primarily discusses the effect of Tongmai Xianxiang′s active ingredients on PHN rather than the effect of the original Chinese herbal base of Tongmai Xianxiang on PHN. The network comprised 263 active ingredients and 53 potential targets, totaling 318 nodes and 1117 edges. The network was sorted based on degree value, highlighting Z‐butylidenephthalide, 2‐undecanol, 1,6,10‐dodecatrien‐3‐OL, 3,7,11‐trimethyl‐, (E)‐, benzene, (1‐butylhexyl)‐, and benzene, (1‐butyloctyl)‐ as potentially key active ingredients for Tongmai Xianxiang′s action against PHN, as shown in Table 1. The figure illustrates that Tongmai Xianxiang exhibits characteristics of multicomponent and multitarget anti‐PHN effects.

**Table 1:** The order of Tongmai Xianxiang′s core active substance.

**Target**	**BC value**	**CC value**	**Degree value**
Z‐Butylidenephthalide	0.006027029	0.481762918	15
2‐Undecanol	0.007688771	0.48030303	14
1,6,10‐Dodecatrien‐3‐ol, 3,7,11‐trimethyl‐, (E)‐	0.009068186	0.478851964	13
Benzene, (1‐butylhexyl)‐	0.003770999	0.478851964	13
Benzene, (1‐butyloctyl)‐	0.003770999	0.478851964	13
Heptanoic acid, ethyl ester	0.00680921	0.477409639	12
Benzene, (1‐butylheptyl)‐	0.00315785	0.477409639	12
Benzene, hexyl‐	0.00315785	0.477409639	12
10,11‐Epoxycalamenene	0.004431551	0.474550898	10
Agarospirol	0.001352239	0.474550898	10
Bicyclo[3.1.1]hept‐2‐ene‐2‐methanol, 6,6‐dimethyl‐	0.001352239	0.474550898	10
Copaene	0.001352239	0.474550898	10
a‐Bisabolol	0.001352239	0.474550898	10
a‐Terpineol	0.001352239	0.474550898	10
*Cis*‐Thujopsene	0.001352239	0.474550898	10

### 3.5. PPI Network Construction and Screening of Core Targets

**Figure 5:**
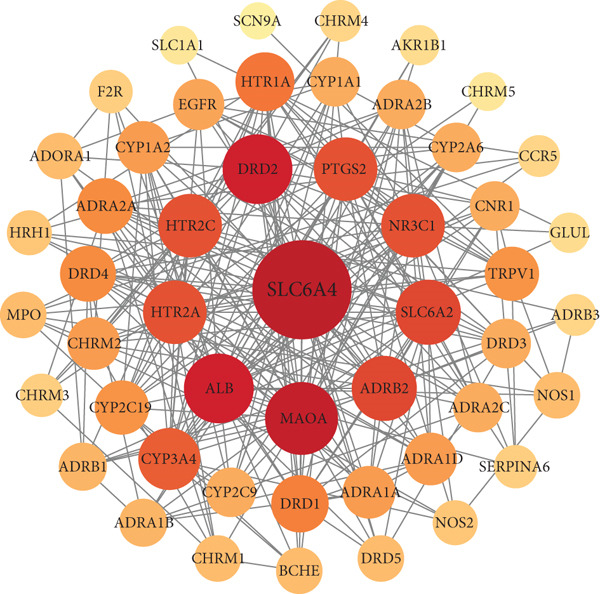
The PPI network diagram. The darker the color of the graph, the stronger the effect of the protein shown.

Cytoscape 3.9.1 was used to generate the PPI network diagram, which includes 50 nodes and 262 edges, as depicted in Figure 5.

The Network Analyzer plug‐in in Cytoscape was utilized to analyze and identify the Top 10 key proteins: SLC6A4, MAOA, DRD2, ALB, ADRB2, SLC6A2, HTR2A, HTR2C, NR3C1, and PTGS2, which play pivotal roles within the network. Sort according to the degree values. SLC6A4, MAOA, DRD2, and ALB are identified as core targets of Tongmai Xianxiang in the treatment of PHN, as shown in Table 2.

**Table 2:** The order of the core proteins.

**Protein**	**Degree**
SLC6A4	25
MAOA	24
DRD2	22
ALB	22
ADRB2	19
SLC6A2	19
HTR2A	18
HTR2C	18
NR3C1	18
PTGS2	18

### 3.6. GO and KEGG Enrichment Analysis Results

GO and KEGG analyses of the 53 potential targets were conducted using the DAVID database, yielding a total of 203 GO entries. These include 118 biological processes, 60 molecular functions, 25 cellular components, and 20 KEGG enrichment pathways.

**Figure 6:**
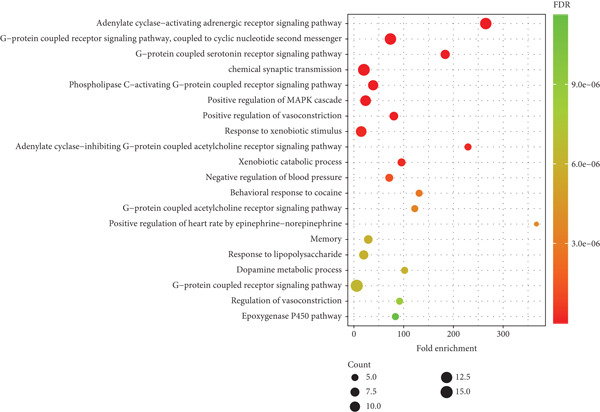
The main biological processes involved in the prevention and treatment of postherpetic neuralgia by traditional Chinese medicine incense (Top 20). The *x*‐axis represents fold enrichment, which is the number of genes enriched above each item.

As depicted in Figure 6, biological processes primarily encompass the adenylate cyclase‐activating adrenergic receptor signaling pathway, G‐protein coupled receptor signaling pathway coupled to cyclic nucleotide second messenger, G‐protein coupled serotonin receptor signaling pathway, chemical synaptic transmission, phospholipase C‐activating G‐protein coupled receptor signaling pathway, and positive regulation of the MAPK cascade, among others.

**Figure 7:**
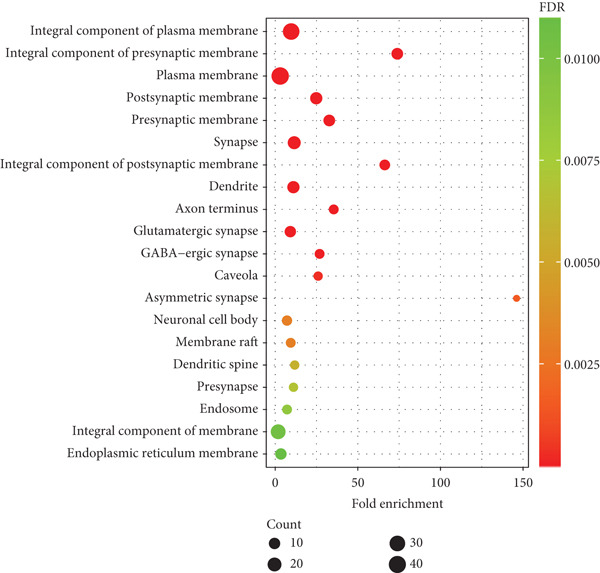
The main cellular components involved in the prevention and treatment of postherpetic neuralgia by traditional Chinese medicine incense (Top 20). The *x*‐axis represents fold enrichment, which is the number of genes enriched above each item.

As depicted in Figure 7, the cellular components primarily include integral components of the plasma membrane, integral components of the presynaptic membrane, plasma membrane, postsynaptic membrane, presynaptic membrane, synapse, integral components of the postsynaptic membrane, dendrite, axon terminus, and glutamatergic synapse.

**Figure 8:**
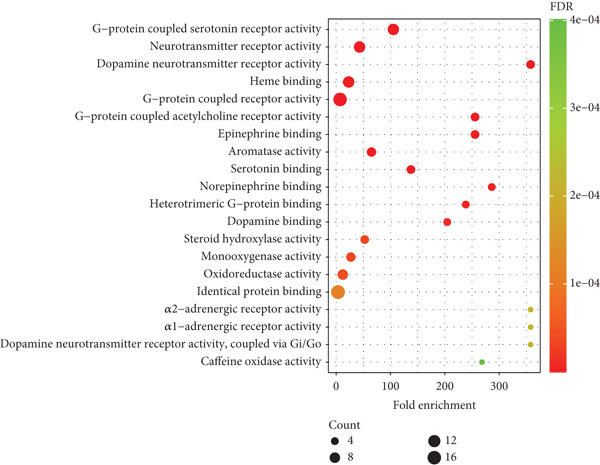
The main molecular functions involved in the prevention and treatment of postherpetic neuralgia by traditional Chinese medicine incense (Top 20). The *x*‐axis represents fold enrichment, which is the number of genes enriched above each item.

As illustrated in Figure 8, the molecular functions primarily include G‐protein coupled serotonin receptor activity, neurotransmitter receptor activity, dopamine neurotransmitter receptor activity, heme binding, G‐protein coupled receptor activity, G‐protein coupled acetylcholine receptor activity, epinephrine binding, aromatase activity, serotonin binding, and norepinephrine binding.

**Figure 9:**
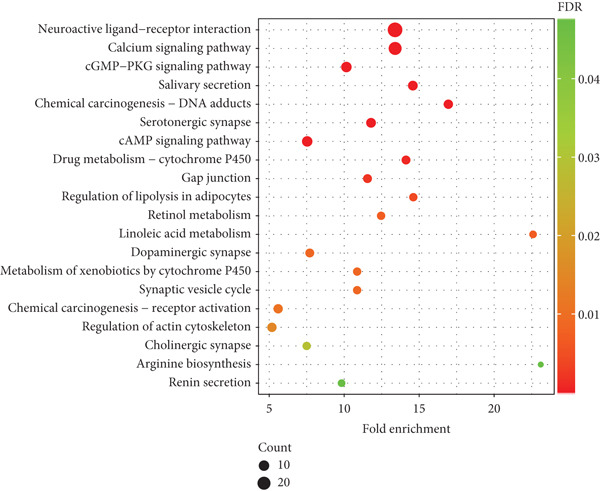
The KEGG pathway enrichment analysis. The *x*‐axis represents fold enrichment, which is the number of genes enriched above each item.

The KEGG enrichment pathways shown in Figure 9 mainly include neuroactive ligand–receptor interaction, calcium signaling pathway, cGMP‐PKG signaling pathway, salivary secretion, chemical carcinogenesis–DNA adducts, serotonergic synapse, cAMP signaling pathway, drug metabolism–cytochrome P450, gap junction, and regulation of lipolysis in adipocytes.

**Figure 10:**
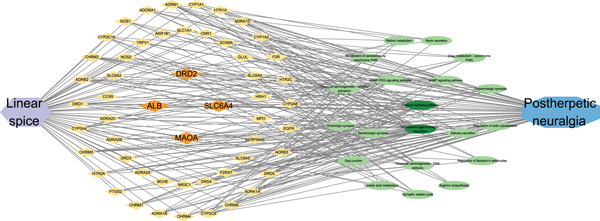
The component–target–pathway–disease network.

As depicted in Figure 10, the component–target–pathway–disease network diagram was created using Cytoscape 3.9.1 software.

### 3.7. Molecular Docking Results

The Top 4 key targets (SLC6A4, MAOA, DRD2, and ALB) in the PPI network were selected for molecular docking verification with the main active ingredients of traditional Chinese medicine: Z‐butylidenephthalide, 2‐undecanol, 1,6,10‐dodecatrien‐3‐ol, 3,7,11‐trimethyl‐, (E)‐, benzene, (1‐butylhexyl)‐, and benzene, (1‐butyloctyl)‐. The results are presented in Table 3.

**Table 3:** The molecular docking results of the effective active components and core targets.

**Core target**	**Main active ingredient**	**Binding energy (kcal/mol)**	**Hydrogen bond numbers**
SLC6A4	Z‐Butylidenephthalide	−5.33	1
	2‐Undecanol	−3.58	2
	1,6,10‐Dodecatrien‐3‐ol, 3,7,11‐trimethyl‐, (E)‐	−5.12	0
	Benzene, (1‐butylhexyl)‐	−5.71	0
	Benzene, (1‐butyloctyl)‐	−5.2	0

MAOA	Z‐Butylidenephthalide	−6.76	1
	2‐Undecanol	−4.27	2
	1,6,10‐Dodecatrien‐3‐ol, 3,7,11‐trimethyl‐, (E)‐	−5.79	2
	Benzene, (1‐butylhexyl)‐	−5.73	0
	Benzene, (1‐butyloctyl)‐	−5.89	0

DRD2	Z‐Butylidenephthalide	−4.93	1
	2‐Undecanol	−3.36	1
	1,6,10‐Dodecatrien‐3‐ol, 3,7,11‐trimethyl‐, (E)‐	−3.11	1
	Benzene, (1‐butylhexyl)‐	−4.21	0
	Benzene, (1‐butyloctyl)‐	−4.32	0

ALB	Z‐Butylidenephthalide	−7.2	1
	2‐Undecanol	−5.32	2
	1,6,10‐Dodecatrien‐3‐ol, 3,7,11‐trimethyl‐, (E)‐	−7.38	1
	Benzene, (1‐butylhexyl)‐	−6.09	0
	Benzene, (1‐butyloctyl)‐	−6.54	0

The results of molecular docking assessed the binding activity between active components of traditional Chinese medicine and core targets. A binding energy ≤ −5 kcal/mol indicated a favorable interaction between the small molecule ligand and protein receptor. Stable interactions, facilitated by hydrogen bonds, were observed between molecules. Based on the lowest binding energies and hydrogen bond formations in molecular docking, the Top 3 combinations with the strongest binding abilities were selected: ALB with Z‐butylidenephthalide, 2‐undecanol, and 1,6,10‐dodecatrien‐3‐ol, 3,7,11‐trimethyl‐ (E)‐, as depicted in Figure 11. Specifically, Z‐butylidenephthalide exhibited strong binding to ALB through a hydrogen bond with LYS414 amino acid residues. 2‐Undecanol formed stable interactions with ALB via two hydrogen bonds involving ASP417 and VAL473 amino acid residues. Similarly, 1,6,10‐dodecatrien‐3‐ol, 3,7,11‐trimethyl‐, (E)‐ interacted closely with ALB through a hydrogen bond with LYS414 amino acid residues.

Figure 11Molecular docking diagram of effective active components and core target proteins: (a) ALB and Z‐butylidenephthalide, (b) ALB and 2‐undecanol, (c) ALB and 1,6,10‐dodecatrien‐3‐ol, 3,7,11‐trimethyl‐, (E)‐.

**Figure:**
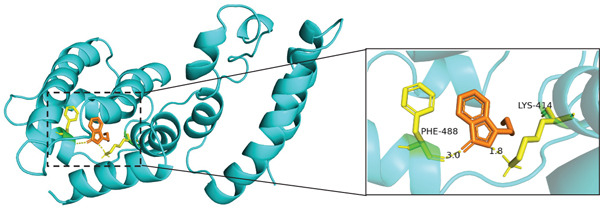
(a)

**Figure:**
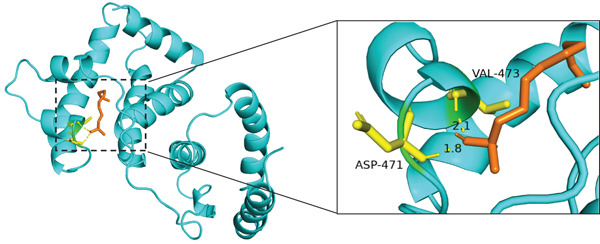
(b)

**Figure:**
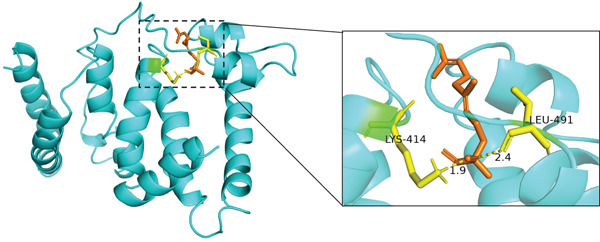
(c)

## 4. Discussion

PHN, the most severe complication of herpes zoster, significantly increases patient pain and compromises their physical and mental health. Current drugs used for PHN treatment often entail substantial side effects and poor patient compliance. Aromatherapy is an alternative treatment method that has proven effective in various clinical scenarios, such as pain. Therefore, integrating nondrug therapies as adjuncts to PHN treatment is crucial.

This paper primarily discusses the mechanism of action of Tongmai Xianxiang in preventing PHN, based on GC‐MS analysis and network pharmacology, providing insights into its actual effects on the human body.

GC‐MS was employed to analyze the volatile components of Tongmai Xianxiang. This study identified 567 substances with well‐defined composition structures and a difference between the actual retention index and the reference retention index < 20. After matching and prioritization using the Swiss Target Prediction database, 772 drug targets were identified.

Through database searches, a total of 169 disease targets related to PHN were identified. The intersection of Tongmai Xianxiang′s targets with PHN‐related targets yielded 53 core targets. According to the PPI network diagram, ALB, SLC6A4, MAOA, and DRD2 emerged as crucial targets for treating PHN.

Molecular docking results indicated that ALB exhibited the strongest binding affinity with Tongmai Xianxiang′s main active compounds, suggesting ALB as its primary target.

ALB is the predominant protein in human serum, as an important nutrient, participating in various physiological and pathological processes [36–39]. Studies [40] have shown that serum ALB levels decrease significantly in patients with herpes zoster, implicating low antioxidant levels in conditions like varicella–zoster virus reactivation and acute nerve injury, potentially leading to PHN.

Z‐Butylidenephthalide binds to the enzyme in a pocket close to the catalytic site, showing antihyperglycemic activity and antitumor effects [41, 42]. Studies have shown that butylidenephthalide exhibited affinity toward 5‐HT (7) receptors in a competitive binding assay [43], and it can also act on calcium ion channels, exerting the effects of a sedative [44, 45], relieving pain [46], and exhibiting significant neuroprotective effects [47].

1,6,10‐Dodecatrien‐3‐ol, 3,7,11‐trimethyl‐, (E)‐ is also called nerolidol. Studies have shown that nerolidol has various functions, such as antioxidant, antitumor, antinociceptive, and anti‐inflammatory properties [48]. It has high hydrophobicity, thereby allowing easier penetration across the plasma membrane and interaction with intracellular proteins, mediates a potent antioxidant activity by scavenging free radicals, preventing lipid peroxidation, and enhancing the production of antioxidant enzymes in cells for protection against oxidative stress [49, 50]. Nerolidol has antinociceptive and anti‐inflammatory activity and also can decrease the levels of polymorphonuclear cells and tumor necrosis factor [51].

2‐Undecanol is currently less used in the medical field and mainly serves as a pheromone component and chemical reagent in biological and medical research [52, 53].

ALB acting as a carrier protein for various endogenous molecules can elevate body antioxidant levels, potentially mitigating pain sensitization and long‐term anxiety associated with PHN [40, 54, 55]. Based on the results of network pharmacology and molecular docking, it is speculated that Z‐butylidenephthalide, 2‐undecanol, and 1,6,10‐dodecatrien‐3‐ol, 3,7,11‐trimethyl‐ (E)‐ can act on ALB through pathways such as neuroactive ligand–receptor interaction, calcium signaling pathway, serotonergic synapse, and anticancer pathways, by the action of appropriately elevating ALB levels to alleviate PHN.

### 4.1. Limitation

Due to time constraints and a lack of a detailed experimental design, this study has not undergone in vivo and in vitro experiments or clinical observations. The findings underscore the necessity for future research. At present, through GC‐MS analysis and network pharmacology analysis, it has been proven that Tongmai Xianxiang can prevent and treat PHN. In the future, we will further carry out relevant clinical research or a dry–wet experiment based on this study.

## 5. Conclusions

Based on GC‐MS analysis and predicted through network pharmacological analysis, Tongmai Xianxiang affects the protein levels mainly including ALB through neural activity ligand–receptor interactions, calcium signaling pathways, 5‐hydroxytryptamine synapses, and anticancer pathways, exerting functions such as antipain, antioxidant, and neuroprotection and regulating the pathological processes related to PHN. This study indicates that Tongmai Xianxiang, through its multicomponents, multitargets, and multipaths of action, can prevent and treat PHN, providing a theoretical basis for clinical applications and laying the groundwork for future research in this area.

## Disclosure

All authors read and approved the final manuscript.

## Conflicts of Interest

The authors declare no conflicts of interest.

## Author Contributions

Linglong Qu and Hui Wang confirmed the authenticity of all the raw data and edited the manuscript. Yufeng Huang, Zhen Gao, and Ruichang Song collected data and processed the data. Shiyu Sun, Pingping Shao, and Rukai Wang conducted the statistics. Chunlin Li reviewed and revised the article.

## Funding

This study was funded by the Shandong Province Technology Innovation Guidance Program (2023luyu‐01) and the Shandong Province Traditional Chinese Medicine Science and Technology Development Program (kt2020039).

## Data Availability

The data that support the findings of this study are available from the corresponding author upon reasonable request.
